# Optimized Antibacterial Effects in a Designed Mixture of Essential Oils of *Myrtus communis*, *Artemisia herba-alba* and *Thymus serpyllum* for Wide Range of Applications

**DOI:** 10.3390/foods11010132

**Published:** 2022-01-05

**Authors:** Wessal Ouedrhiri, Hamza Mechchate, Sandrine Moja, Sylvie Baudino, Asmaa Saleh, Omkulthom M. Al Kamaly, Andriy Grafov, Hassane Greche

**Affiliations:** 1 Laboratory of Inorganic Chemistry, Department of Chemistry, University of Helsinki, P.O. Box 55, FI-00014 Helsinki, Finland; Andriy.grafov@helsinki.fi; 2 Université de Lyon, UJM-Saint-Etienne, CNRS, BVpam, FRE3727, F-42023 Saint-Etienne, France; Sandrine.moja@univ-st-etienne.fr (S.M.); Sylvie.baudino@univ-st-etienne.fr (S.B.); 3 Department of Pharmaceutical Sciences, College of Pharmacy, Princess Nourah Bint Abdulrahman University, P.O. Box 84428, Riyadh 11671, Saudi Arabia; ASAli@pnu.edu.sa (A.S.); omalkmali@pnu.edu.sa (O.M.A.K.); 4 National Agency of Medicinal and Aromatic Plants, University Sidi Mohamed Ben Abdellah, Taounate BP 159, Morocco; hgreche@yahoo.fr

**Keywords:** essential oil, antibacterial, mixture design, combination, microdilution, optimal mixture

## Abstract

Nowadays, the combination of molecules influences their biological effects, and interesting outcomes can be obtained from different component interactions. Using a mixture design method, this research seeks to simulate the efficacy of essential oil combinations against various bacteria and forecast the ideal combination. The chemical compositions of *Myrtus communis*, *Artemisia herba-alba* and *Thymus serpyllum* essential oils were analyzed using CG/MS. Then, the combined antibacterial effects were evaluated by testing mixture design formulations using the microdilution bioassay. The main compounds detected for *M. communis* essential oil were myrtenyl acetate (33.67%), linalool (19.77%) and 1,8-cineole (10.65%). *A. herba-alba* had piperitone as a chemotype, representing 85%. By contrast, the *T. serpyllum* oil contained thymol (17.29%), γ-terpinene (18.31%) and p-cymene (36.15%). The antibacterial effect of the essential oils studied, and the optimum mixtures obtained were target strain-dependent. *T. serpyllum* alone ensured the optimal inhibition against *S. aureus* and *E. coli*, while a ternary mixture consisting of 17.1%, 39.6% and 43.1% of *M. communis*, *A. herba-alba* and *T. serpyllum* respectively, was associated with optimal inhibitory activity against *B. subtilis*. The outcome of this research supports the idea of the boosting effect of essential oil combinations toward better activities, giving better understanding of the usefulness of mixture designs for food, cosmetics, and pharmaceutical applications.

## 1. Introduction

Aromatic and volatile oily substances derived from plant material are known as essential oils. Their usage as environmentally acceptable alternatives to combat food-borne germs and other harmful microbes is becoming more popular across the globe [1,2]. In fact, several studies have evaluated their effectiveness against food-borne bacteria, and several essential oils have been tested in cheese [3], meat [4], and rice [5].

Nowadays, some of the crude essential oils from thyme, oregano, cinnamon and others have been classified as GRAS (generally recognized as safe) by the FDA (United States Food and Drug Administration). Likewise, several compounds including limonene, eugenol, carvacrol, and thymol have been accepted as usable in food by the European Commission [6]. However, studies on essential oils interactions with the food matrix have shown ineffective outcomes [7]. In fact, high concentrations are needed to achieve a similar antibacterial (Higher that those obtained with in vitro assays) [6,8] which can lead to an outstripping of their toxic doses and an alteration of the organoleptic quality of food [8,9,10].

Therefore, many researchers studying the antibacterial potential of essential oils and focusing on their combinations and interactions, have attempted to benefit from their synergistic outcomes, and improve their efficiency, lowering the effective dosage [7]. Generally, the mode of action of antimicrobial essential oils influences their combinations. In fact, the volatile compounds present either in a single essential oil or in a mixture of oils affect the target bacteria’s various biochemical processes, and produce various interactive antibacterial effects [8]. Synergistic and additive antibacterial effects have been observed between numerous essential oils, such as *Origanum vulgare* and *Rosmarinus officinalis* [11], *Lippia multiflora* and *Mentha piperita* [12], *Cymbopogon citratus* and *Cymbopogon giganteus* [13].

In this study, three essential oils were selected for evaluation of their combined antibacterial effect. First, *M. communis* L. or myrtle belonging to the *Myrtaceae* family which has been used since ancient times as a spice, medicinal agent, and in food preparation purposes. Its antibacterial, antifungal, and antioxidant effects have been exploited in food preservation [14]. Second, *A. herba-alba* or wormwood belonging to *Asteraceae* family, which has been used in Morocco as herbal teas in folk medicine [15] and known for its antibacterial and antioxidant effects [16,17]. Third, *T. serpyllum* L. or wild thyme belonging to *Lamiaceae* family, which has been utilized in foreign cuisine and by locals for a variety of purposes, including antibacterial, anthelmintic, carminative, sedative, and as a tonic [18].

To assess the antimicrobial interactions between essential oils, this study coupled the mixture design approach with the standardized microdilution method in order to determine the minimum inhibitory concentration of different mixtures [19]. Contrary to the checkerboard and time-kill tests [9,20,21,22], modeling by mixture design gives a general conception of the response of all possible combinations and defines the interactions between factors [23]. Thus, the mixture of essential oils giving the optimal response can be easily defined [24].

Different studies have used mixture design to obtain the best mixture of a given extract or molecules. In a recent study, the mixture of rutin, catechin, and epicatechin was evaluated for an optimum antidiabetic formulation [25], and the mixture of plant extract prepared from *Olea europaea*, *Coriandrum sativum* and *Linium usitatisimum* was assessed for an optimum antihyperglycemic formulation [26]. In contrast, in another study, *Myrtus communis*, *Pimpinella anisum*, and *Carum carvi* plant extract mixture was studied for an optimum formulation to treat intestinal comfort [27].

Our study used this experimental methodology in order to contribute to the development of essential oil preservatives in food. The objectives were to evaluate the antibacterial combined effect of Moroccan *M. communis*, *A. herba-alba*, and *T. serpyllum* essential oils and to determine the optimal inhibitory effect against different bacterial strains alone and against a combination of all of the strains studied.

## 2. Materials and Methods

### 2.1. Plant Material and Essential Oil Extraction

Aerial parts of *M. communis* (NAMAP-XP45) and *T. serpyllum* (leaves and stems) (NAMAP-XP95), and *A. herba-alba* (leaves, stems and flowers) (NAMAP-XP101) were harvested in June, July, and August 2012, respectively, from Taounate region (Morocco). The plants were identified by taxonomist Abdeslam Ennabili. Voucher specimens of each plant were deposited in the herbarium of the National Agency of Medicinal and Aromatic Plants (NAMAP), Morocco.

The fresh aerial parts (leaves and stems) of each plant were hydrodistillated for three hours using a Clevenger apparatus (VWR, Radnor, Pennsylvania, United States to extract essential oils (100 mg of powdered plant extract added to 500 mL of distillated water in a 2 L round-bottom flask). The essential oils were kept in the dark and at 4 °C until they were needed.

### 2.2. Gas Chromatography-Mass Spectrometry (GC/MS) Analysis Conditions

The essential oil GC/MS analysis was performed using an Agilent GC-MSD system (Agilent Technologies 6850/5973, Santa Clara, CA, USA) with helium (high purity) as the carrier gas at a constant linear velocity of 36 cm/s. Operating at 70 eV ionization energy and scanning the m/z range of 50–550, the transfer, source, and quadrupole temperatures were 245 °C, 230 °C, and 150 °C, respectively. An Agilent DB5 ms capillary column (30.0 m 0.25 mm 0.25 m film thickness) was employed, which was set from 60 °C to 245 °C at 3 °C/min. Hexane was used to dilute the essential oil samples (1:3000). In splitless mode, the injection volume was 2.0 L, and the injector temperature was 250 °C. Individual components were identified by comparing mass spectra of real reference compounds when feasible, as well as referring to the NIST MS Search database 2012 and the Adams terpene library [28].

### 2.3. Antibacterial Activity Assessment

#### 2.3.1. Agar-Disc Diffusion Test

Four bacterial strains were selected to evaluate the essential oil activity, namely *Bacillus subtilis* (ATCC 3366), *Pseudomonas aeruginosa* (ATCC 27853), *Staphylococcus aureus* (ATCC 29213), and *Escherichia coli* (ATCC 25922). An agar disc diffusion assay was carried out, as recommended by CLSI [29], with some modifications. To inoculate Mueller Hinton Agar plates, fresh bacterial solution prepared in sterile saline and adjusted to 0.5 McFarland was utilized (Biokar diagnostics, Beauvais, France). Then, on the surface of each plate, sterile paper discs (6 mm in diameter) were placed and soaked with 10 microliters of essential oil. Plates were incubated at 4 °C for 2 h before being moved to 37 °C for 18–24 h. Inhibition zone diameters were measured in millimeters. All of the experiments were conducted three times.

#### 2.3.2. Determination of Minimum Inhibitory Concentration (MIC)

The microdilution technique was used to determine the minimal inhibitory doses of essential oils. It was carried out following a modified version of El Karkouri et al. protocol [30]. In this test, two-fold serial dilutions of the essential oils ranging from 4 to 0.031% (*v*/*v*) were made in Mueller Hinton Broth (MHB), supplemented with 0.15 % (*w*/*v*) of bacteriological agar (Biokar diagnostics, Beauvais, France). Then, at a final concentration of 106 CFU/mL, the bacterial suspension (made in the same medium) was applied to each well. The microplates were then incubated for 18 h at 37 °C. To determine active bacterial growth, 10 µL of resazurin was applied to each well after the incubation time. After a second incubation at 37 °C for 2 h, the lowest EO concentration that induces a change of resazurin color was taken as MIC. The conversion of the blue dye resazurin to pink resorufin revealed active microbial growth. All of the experiments were carried out three times.

#### 2.3.3. Determination of Minimum Bactericidal Concentration (MBC)

The least amount of essential oil causing a negative subculture corresponds to the MBC value, and it is calculated after the following: in prepared Luria Bertani plates, 5 µL from negative wells are spread and incubated for 24 h at 37 °C [19].

### 2.4. Mixture Design and Statistical Analysis

An augmented simplex-centroid design was utilized to evaluate the ternary antibacterial efficacy of the tested EOs [24]. The factors reflect the proportion of each essential oil in combination, which may vary from 0 to 1 with no design limits.

**Figure 1:**
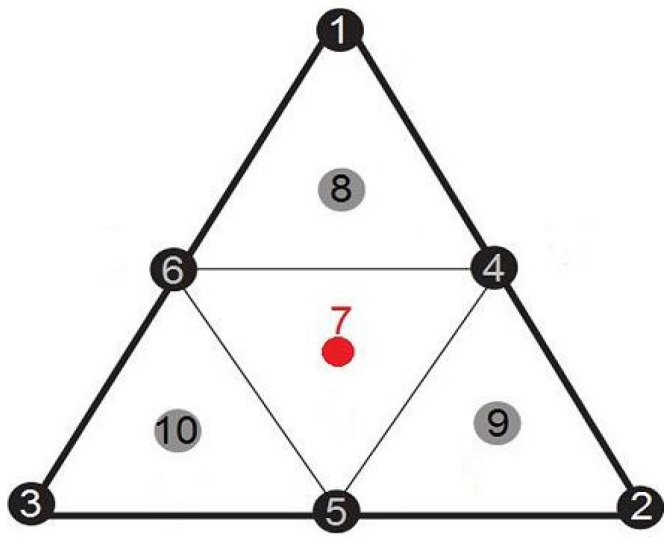
Experiments to be carried out in a centered augmented mixture design.

The points of the intended tests are shown in Figure 1. The three essential oils are represented by the triangle’s vertices (1, 2, 3). The binary combinations and the central point (centroid) and the three augmented points (8, 9, 10) allocated to the ternary combinations are represented by the midpoints of the three sides of the triangle (4, 5, 6). Section 3.3.1 shows that the whole experimental design for each bacterial strain included 18 trials, including three replications of the center point. The same section shows the sequence in which the trials were carried out.

The antibacterial properties against *B. subtilis* ATCC 3366, *S. aureus* ATCC 29213, and *E. coli* ATCC 25,922 were tested using the microdilution technique described in Section 2.3.2.

Afterwards, the data were fitted to a special cubic polynomial model applying the least squares regression to estimate the unknown coefficients in Equation (1):

(1) $$Y={\;b}_{1}X_{1}+b_{2}X_{2}+b_{3}X_{3}+b_{12}X_{1}X_{2}+b_{13}X_{1}X_{3}+b_{23}X_{2}X_{3}+b_{123}X_{1}X_{2}X_{3}$$

where Y is the response, b_i_ is the magnitude of each component’s influence X_i_, b_ij_ is the magnitude of the interaction effect of two components on the response, and b_ijk_ is the magnitude of the interactive effect of three components on the response. The proportions of the component I in the mixture are denoted by X_i_. The SAS JMP software, version 8.0.1, was used for this investigation.

## 3. Results and Discussion

### 3.1. Essential Oils Composition

The essential oils from aerial parts of *M. communis*, *A. herba-alba* and *T. serpyllum* were obtained with yields of 0.5%, 1% and 0.9% (*v*/*w*), respectively.

Table 1 describes the forty-one components accounting for 98.59% of the total amount of *M. communis* EO. The major compounds obtained were myrtenyl acetate (33.67%), linalool (19.77%), 1,8-cineole (10.65%), and limonene (8.96%). Few of the previously described chemical profiles corroborate this finding [31]. Most authors have found α-pinene as the major compound [32,33,34,35], and sometimes 1,8-cineole [36], or linalool [37].

As regards *A. herba-alba*, nineteen compounds accounting for 94.9% of the total oil were identified and the major compound was piperitone (85.86%) (Table 1).

Considering the major constituents, the literature reveals that essential oils of wormwood exhibit intraspecific variation of its chemical composition, thus several chemotypes are known for this oil. Tisserand et al. have reported 7 chemotypes of *A. herba alba* essential oil, dividing them into those predominated by one compound, and those predominated by two or three compounds [38]. The chemotypes most encountered were the ones dominated by thujone which may reach up to 95% [38] of α-thujone [39] and β-thujone [40]. Other studies reported camphor [40], chrysanthenone, chrysanthenyl acetate [41], davanone, pinocarvone [42], α-thujone/camphor, α-thujone/β-thujone, and 1,8-cineole/camphor/thujone (α + β) [16] chemotypes. However, no piperitone chemotype has been previously found. This polymorphism observed between chemical compositions can be related to several causes as reported in published data, such as sampling origin, mineral amount in soil (Zn, Cu, Fe, Mn), mean monthly temperature and precipitation values. Thus, the chemovariation might also be genetically determined [43,44].

For *T. serpyllum* essential oil, its chemical composition was reported in our previous study [45]. Briefly, the major compounds were p-cymene (36.15%), γ-terpinene (18.31%) and thymol (17.29%).

All 3 essential oils GC/MS chromatogram are provided in the Supplementary File (Figures S1–S3).

### 3.2. Single Antibacterial Effect

As shown in Table 2, wild thyme demonstrated the most marked antibacterial effect with a MIC and MBC from 0.125 to 0.5%, while *M. communis* and *A. herba alba* demonstrated MIC and MBC values of 0.125 and up to 4%. As for *P. aeruginosa* only thyme demonstrated MIC and MBC values of 4%. This strain was not susceptible to myrtle and white wormwood oils. *P. aeruginosa* was the most resistant strain tested, whereas *B. subtilis* was the most susceptible.

Previous studies have reported on the antibacterial efficacy of the EOs studied in this research. Most of these studies have always linked this activity to their chemical composition. For example, Tunisian *M. communis* essential oil with a chemical composition similar to that found in this study, i.e., rich in myrtenyl acetate (20.75%), 1,8-cineole (16.55%), α-pinene (15.59%), and linalool (13.30%), showed an inhibitory effect against *P. aeruginosa*, *E. coli*, *S. aureus*, *Listeria monocytogenes*, *B. subtilis* and *Salmonella enteritidis* [46]. Nevertheless, this result could be explained by several other hypotheses, such as the quantity of monoterpenes known for their antibacterial effect. The properties of linalool, 1,8-cineole, and limonene have been established [10] and some oxygenated monoterpenes (carvacrol) are able to affect cellular integrity [46].

The wormwood essential oil studied, rich in piperitone (85%), exhibited moderate antibacterial activity. However, some studies focused on the antibacterial activity of *A. herba-alba* essential oil confirmed its efficiency against several pathogenic strains [16,47]. Moreover, a study on *Achillea biebersteini* fraction, with 82% of piperitone showed moderate antimicrobial activity compared to other fractions [48]. Nevertheless, a high fungicidal effect of piperitone has been reported [49].

*T. serpyllum* essential oil had a substantial inhibitory effect, which might be explained by the presence of γ-terpinene and thymol, both of which have previously been identified as bactericidal substances [50]. Thus, the synergistic effect that might be produced by the interaction of thymol with γ-terpinene and p-cymene working as hydrocarbon monoterpenes is explained by the fact that they facilitate the penetration of thymol due to their hydrophobic character as has been previously reported [9]. Indeed, p-cymene and thymol have already shown a synergistic antifungal effect [51]. Furthermore, the three major compounds of *T. serpyllum* have the same mechanism of action against bacteria; in fact, they decrease the lipid melting temperatures of membranes and increase membrane fluidity [10].

### 3.3. Ternary Combination and Its Antimicrobial Action

Our recent work [45] has shown that the formulation of essential oils based on the augmented simplex-centroid mixture design was more rational than the prediction of the antibacterial combined effect using the standardized antibacterial method. This new strategy has been thus applied here to define the mixture of *M. communis*, *A. herba-alba* and *T. serpyllum* essential oils giving an optimal antibacterial effect.

#### 3.3.1. Establishment of the Response Prediction Model

For each strain, the experimental data were utilized to create a prediction model (Table 3 and Figures S4–S6 in Supplementary File). From the results, the activity difference of each single and combined essential oil it can be seen. *T. serpyllum* single application has the best inhibition against *S. aureus* and *E. coli* with a MIC % of 0.125 and 0.250; while against *B. subtilis* the best MIC was obtained with the ternary mixture of essential oils (0.125%). The results indicate that the single application of an essential oil can only be effective against some strains and the combination of multiple essential oils can boost their effect to inhibit a wide range of microbes. The special cubic models were selected to describe the link between the response variables and the factors (Table 4). The modified R2 values and the coefficient of determination (R2) values suggested that the chosen models offered a satisfactory fit to the data (Table 4). Furthermore, the polynomial regression models’ statistical significance was shown using analysis of variance (ANOVA) (Table 4).

Generally, in the model obtained, each coefficient reflects the magnitude of the antimicrobial effect of its associated factor. Accordingly, a coefficient with a low value in the fitted model reflects the capacity of its associated factor to decrease the response (MIC value), which consequently means the capacity to increase the antibacterial effect. The coefficients with high values show the capacity of their associated factors to increase the response.

#### 3.3.2. Model Validation

To confirm the validity of the selected model, we used the point-test. We choose an arbitrary formula and we test its antibacterial activity, then we compare it to the predicted value. The coordinates of the test point chosen for Gram positive strains are: X*_M. communis_* = 0.9%, X*_A. herba-alba_* = 0.05% and X*_T. serpyllum_* = 0.05%. The MIC obtained with this formulation was 0.5 and 1 respectively for *B. subtilis* and *S. aureus*. Values very close to those calculated by the mathematical model of each strain were 0.43% and 0.96% respectively.

Regarding the coordinates of the test point chosen for *E. coli* are: X*_M. communis_* = 0.1%, X*_A. herba-alba_* = 0.1% and X*_T. serpyllum_* = 0.8%. The MIC obtained experimentally, which is equal to 1%, does not differ from that calculated mathematically (0.98%).

#### 3.3.3. The Influence of Mixture Components and Their Interactions on Responses

The volatile compounds of the essential oils or their mixtures may interact, thereby producing four possible types of interactive outcomes: indifference, additivity, antagonism, and synergy. These interactions, which are certainly influenced by the chemical composition of each essential oil, could decrease or increase the antimicrobial efficiency of the whole mixture [9].

**Figure 2:**
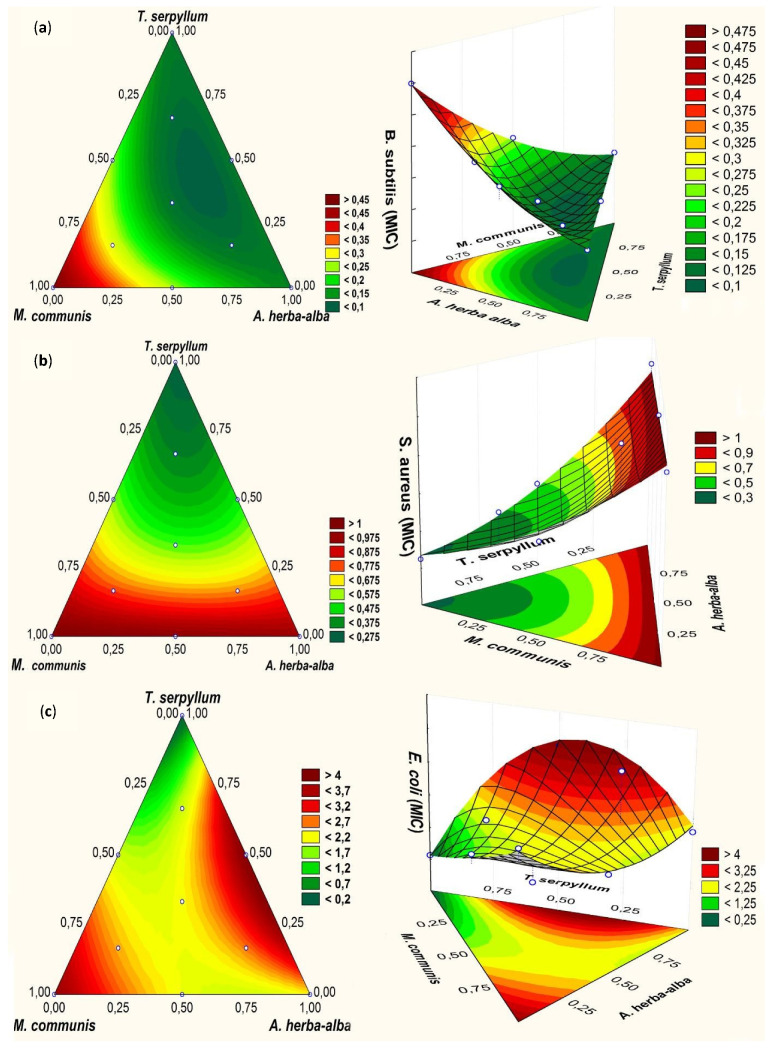
The 2D contour plot (A) and 3D surface plot (B) based of the obtained MIC. (**a**): *B. subtilis*; (**b**): *S. aureus*; (**c**): *E. coli*.

The 2D contour plots and 3D surface plots of the responses (MICs) for *B. subtilis*, *S. aureus*, and *E. coli* are shown in Figure 2.

Following model equations, *T. serpyllum* oil had the weakest coefficients (Table 5), followed by that of *A. herba-alba* and then *M. communis*, which confirms the strength of their antibacterial effect previously discussed. A significant synergistic effect was obtained from the binary interactions of myrtle/wild thyme and myrtle/wormwood against *B. subtilis* (*p* < 0.01). Moreover, only the binary interaction of myrtle/wormwood was significantly synergistic against *E. coli* (*p* < 0.05). Nonetheless, no significant contribution of the binary interactions against *S. aureus* was noticed (*p* > 0.05). Conversely, wormwood and wild thyme essential oils were suspected to have a significant antagonistic effect against *E. coli* (*p* < 0.001), while the same interaction was not significant against *B. subtilis* (*p* > 0.05) (Table 5). In addition, the ternary interaction was only significantly synergistic against *B. subtilis* (*p* < 0.001).

In fact, the major compounds of myrtle oil were 1,8-cineole and linalool, while p-cymene, γ-terpinene and thymol predominated in thyme oil. This could provide a possible explanation for the synergistic effect obtained by their combination, and explains their effect against *B. subtilis.* It has been reported that the combination of thymol/linalool and thymol/1,8-cineole against several strains produced a synergistic antibacterial effect [51,52]. Furthermore, the combination of 1,8-cineole with hydrocarbon monoterpenes such as limonene and aromadendrene, has already shown additive or synergistic effects [53,54]. The combination of 1,8-cineole and p-cymene, have already shown an antifungal synergistic effect [51].

Regarding myrtle and wormwood essential oils, oxygenated monoterpenes (myrtenyl acetate, 1,8-cineole, linalool, and piperitone) predominated in both of their chemical compositions. Hence, their combination could increase the ratio of oxygenated monoterpenes and could be more effective in producing a synergistic effect against *E. coli* and *B. subtilis*. Nazzaro et al. have reported the antimicrobial activity of most terpenoids to their functional groups, such as hydroxyle and carboxyle, which may affect different sites in bacterial cells [55].

The antagonistic effect obtained by the thyme/wormwood interaction against *E. coli*, could be better explained using Figure 2. It is observed that MIC increases with the increasing wormwood amount in the total mixture. This could reduce the ratio of p-cymene, γ-terpinene and thymol, which usually work in synergy and have the same mechanism of action (i.e., a decrease in the lipid melting temperatures of model membranes, which is suggestive of increased membrane fluidity) [10]. Moreover, according to the MBC interpretation, it was shown that wormwood had a bacteriostatic effect against *E. coli*, while thyme had a bactericide effect. Their combination can support the hypothesis that antagonism occurs when bacteriostatic and bactericidal antimicrobials are combined [6].

The coefficients determined in the interaction between the three essential oils revealed a strong synergistic impact against *B. subtilis* (*p* < 0.001). The ideal zone in the mixed area is located in the middle of the triangle, indicating that there is a good interaction (Figure 2). This interaction did not demonstrate any significant contribution against *S. aureus* and *E. coli*. In this ternary combination, the optimal zone is situated in the triangle base; this means that the amounts of thyme and wormwood essential oils are considerable compared to the myrtle one. However, the coefficient of binary interaction of wormwood/thyme was not significant before, while with the presence of myrtle oil, a significant coefficient was obtained. Indeed, several published data have shown the importance of some minor essential oil compounds. Delaquis et al. [56] demonstrated that essential oil of dill, cilantro or coriander were more effective than their fractions, and the best effect was observed with crude oil. It was also demonstrated that some fractions were better than their crude oils. Hence, attention should be paid to minor components, which are likely to contribute to the outcomes of oil interactions [56].

#### 3.3.4. Mixture Optimization

**Figure 3:**
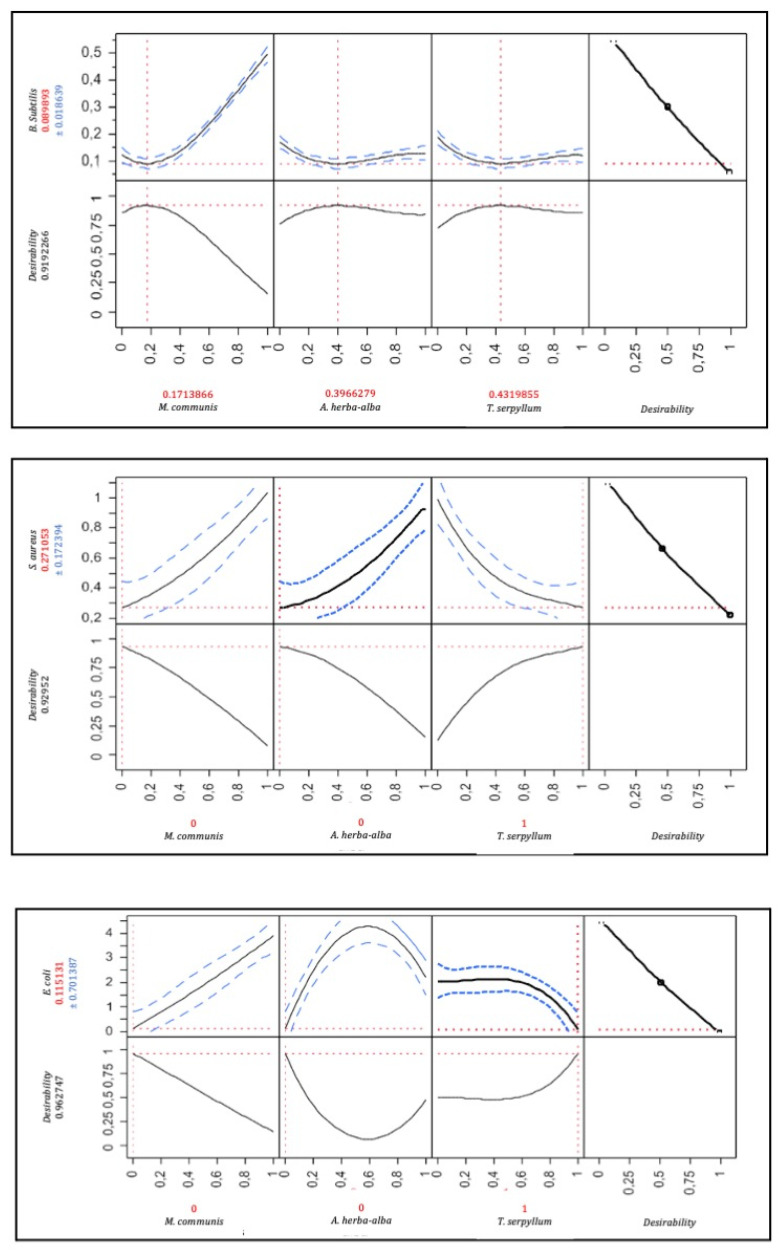
Optimal mixture for each strain and its corresponding desirability.

The optimal mixture specific to each strain and their common optimum were defined using a response optimizer. The optimal mixtures were defined in the optimal zone found for each strain studied (Figure 3). The optimums were specific to each strain, even for both Gram-positive strains, *B. subtilis* optimum corresponded to a mixture containing 17.1%, 39.6% and 43.1% of myrtle, wormwood, and wild thyme, respectively, with MIC value of 0.089 ± 0.018%. Against *S. aureus*, the maximal antibacterial effect was found with 100% of wild thyme, with an MIC value of 0.27 ± 0.17%. Likewise, the Gram-negative strain represented by *E. coli* needed the addition of wild thyme to be inhibited optimally, with a predictive MIC value of 0.11 ± 0.7%. As for the common optimal mixture, the optimizer suggested wild thyme for all strains with MIC values of 0.11%, 0.27% and 0.11% against *B. subtilis*, *S. aureus* and *E. coli*, respectively. In summary, a target strain-dependence was observed in this study, which could be explained by several attack mechanisms specific to each strain.

In addition, optimal mixtures can be used in different industrial domains, in food and even in cosmetics, soap, shampoo, cleaning gels, or pharmaceuticals, for example.

#### 3.3.5. Test-Point

To finalize the validity tests of the chosen model, we used the test point tool. We conducted a test that matched the desired response. The coordinates of the test point chosen for the Gram positive strains are: X*_M. Communis_* = 0.9%, X*_A. herba-alba_* = 0.05% and X*_T. serpyllum_* = 0.05%. The MIC obtained with this formulation in vitro was 0.5 and 1 for *B. subtilis* and *S. aureus,* respectively. Values very close to those calculated by the mathematical model of each strain were 0.43% and 0.96% respectively. The coordinates of the test point chosen for *E. coli* were: X*_M. communis_* = 0.1%, X*_A. herba-alba_* = 0.1% and X*_T. serpyllum_* = 0.8%. The MIC obtained experimentally, and which is equal to 1%, does not differ much from that calculated mathematically (0.98%). The results obtained in this test point validate the mathematical model obtained in this study.

## 4. Conclusions

The present study showed that the optimum was specific for each strain, which makes it difficult to speak about a common optimum mixture. However, the choice of a common effective mixture is still feasible. Furthermore, the less susceptible strains *E. coli* and *S. aureus* were optimally inhibited by wild thyme, while *B. subtilis* was inhibited by the combination of the three essential oils. Therefore, the obtained results confirm that no general relationship between the Gram strain and antibacterial activity could be established for the essential oils. Thus, mixture recipes must not be used traditionally, but studied and modeled according to the target pathogen. Henceforth, these results might aid in the effective use of essential oils as natural preservatives in food or in the cosmetic industries.

## Figures and Tables

**Table 1:** Chemical composition of *M. communis* and *A. herba-alba* essential oils.

*M. communis*				*A. herba-alba*			
Compounds	Rt ^a^	RI ^b^	Peak Area %	Compounds	Rt ^a^	RI ^b^	Peak Area %
α-Thujene	5.28	924	0.16	1R-α-Pinene	5.48	931	0.1
α-pinene	5.48	931	7.17	β-Myrcene	6.96	987	0.09
β-pinene	6.69	977	0.13	*p*-cymene	8.13	1023	1.31
δ-3-carene	7.61	1009	0.15	D-Limonene	8.28	1028	0.2
*o*-cymene	8.13	1024	1.05	β-Thujene	8.35	1029	0.04
Limonene	8.29	1028	8.96	Lavender lactone	8.51	1034	0.12
**1,8-cineol**	**8.41**	**1032**	**10.65**	γ-Terpinene	9.29	1056	0.14
*cis*-Linalool oxide	9.75	1070	0.27	3-Carene	10.84	1099	0.11
*trans*-Linalool oxide	10.34	1087	0.25	*cis*-4-Thujanol	12.55	1141	0.14
**linalool**	**10.87**	**1103**	**19.77**	Fenchone	13.39	1162	0.43
Terpinene-4-ol	14.13	1180	0.2	Terpinen-4-ol	14.12	1179	0.68
*p*-Cymen-8-ol	14.42	1187	0.09	Criptone	14.37	1185	0.23
*cis*-3-hexenyl butanoate	14.48	1188	0.12	(A)-Terpineol	14.75	1195	1.77
β-fenchyl alcohol	14.75	1195	3.1	**Piperitone**	**17.26**	**1254**	**85.68**
Estragol	14.85	1197	0.34	Copaene	22.28	1372	0.15
L-carvone	16.76	1242	0.09	(-)-Spathulenol	30.32	1572	0.15
Citral	17.78	1266	0.1	(+)-Spathulenol	30.32	1572	0.12
**Myrtenyl acetate**	**20.12**	**1321**	**33.67**	Davanone	30.44	1575	3.12
Neryl acetate	21.62	1356	0.18	Monoterpene hydrocarbon			1.99
Methyl eugénol	23.37	1398	0.96	Oxygenated monoterpenes			88.7
*trans*-Caryophyllene	24.06	1415	0.22	Sesquiterpenes hydrocarbon			0.15
α-Humulene	25.52	1451	0.42	Oxygenated sesquiterpenes			3.38
4,6-diethyl-2-methopyrimidine	27.91	1510	1.35	Other			0.35
2-(1,1-Dimethylethyl) phenol	28.56	1527	0.18	Total			94.58
Caryophyllene Oxide	30.48	1576	0.32				
Humulene-1,2-epoxide	31.56	1604	0.41				
Monoterpene hydrocarbon			17.62				
Oxygenated monoterpenes			35.16				
Sesquiterpenes hydrocarbon			0.64				
Oxygenated sesquiterpenes			0.73				
Other			36.15				
Total			90.3				

^a^: Retention time on DB-5 capillary column in minutes; ^b^: Retention index relative to n-alkanes on DB-5 capillary column.

**Table 2:** Antibacterial activity of *M. communis*, *A. herba-alba* and *T. serpyllum* essential oils.

Strains	Inhibition Zone * (mm)			MIC/MBC (% (*v*/*v*))		
	*M. communis*	*A. herba-alba*	*T. serpyllum*	*M. communis*	*A. herba-alba*	*T. serpyllum*
*S. aureus*	17.33 ± 1.52	12.16 ± 0.28	36.00 ± 1.73	1/>2	1/>2	0.25/0.25
*B. subtilis*	17 ± 1	11 ± 1	33.00 ± 2.64	0.5/>2	0.125/>2	0.125/0.5
*E. coli*	11.5 ± 0.5	11 ± 1.73	21.66 ± 2.08	4/>4	2/>4	0.125/0.25
*P. aeruginosa*	-	-	10.00 ± 1.73	-	-	>4/>4

* Disc diameter (6 mm) included.

**Table 3:** EOs proportion in each experiment and experimental responses (MICs).

N° Exp	Mc	Ah	Ts	MIC % (*v*/*v*)		
				*B. subtilis*	*S. aureus*	*E. coli*
1	0.333333	0.333333	0.333333	0.1250	0.500	2.000
2	0.000000	1.000000	0.000000	0.1250	1.000	2.000
3	0.000000	0.500000	0.500000	0.1250	0.500	4.000
4	0.500000	0.000000	0.500000	0.2500	0.500	2.000
5	0.166667	0.666667	0.166667	0.1250	0.500	4.000
6	0.000000	0.000000	1.000000	0.1250	0.250	0.125
7	0.666667	0.166667	0.166667	0.2500	1.000	2.000
8	0.500000	0.500000	0.000000	0.2500	1.000	2.000
9	1.000000	0.000000	0.000000	0.5000	1.000	4.000
10	0.166667	0.166667	0.666667	0.0625	0.500	2.000
11	0.000000	1.000000	0.000000	0.1250	1.000	2.000
12	0.000000	0.500000	0.500000	0.1250	0.500	4.000
13	0.500000	0.000000	0.500000	0.2500	0.500	2.000
14	0.000000	0.000000	1.000000	0.1250	0.250	0.125
15	0.500000	0.500000	0.000000	0.2500	1.000	2.000
16	1.000000	0.000000	0.000000	0.5000	1.000	4.000
17	0.333333	0.333333	0.333333	0.1250	0.500	2.000
18	0.333333	0.333333	0.333333	0.1250	0.500	2.000

Mc: *M. communis*, Ah: *A. herba-alba* and Ts: *T. serpyllum*.

**Table 4:** Responses analysis of variance.

Model	*B. subtilis*					*S. aureus*					*E. coli*				
	df	SS	R^2^	R^2^_adj_	*p*	df	SS	R^2^	R^2^_adj_	*p*	df	SS	R^2^	R^2^_adj_	*p*
Linear	2	0.220407	0.8237	0.8002	0.0000	2	1.106061	0.8044	0.7783	0.0000	2	6.86364	0.2761	0.1796	0.0887
Quadratic	5	0.254783	0.9522	0.9323	0.0000	5	1.210721	0.8805	0.8307	0.0000	3	21.58313	0.8681	0.8132	0.0001
Special cubic	6	0.264001	0.9866	0.9793	0.0000	6	1.234868	0.8981	0.8425	0.0001	6	22.54154	0.9067	0.8558	0.0000
Residual error	11	0.003577	-	-	-	11	0.140132	-	-	-	11	2.31957	-	-	-
Total	17	0.267578	-	-	-	17	1.375000	-	-	-	17	24.86111	-	-	-

**Table 5:** *p*-value and coefficients of each model fitted.

	*B. subtilis*		*S. aureus*		*E. coli*	
	Estimation	*p*-Value	Estimation	*p*-Value	Estimation	*p*-Value
Mc	0.49720	0.000000 ***	1.03355	0.000000 ***	3.8964	0.000000 ***
Ah	0.12845	0.000001 ***	0.95855	0.000000 ***	2.1964	0.000026 ***
Ts	0.11908	0.000001 ***	0.27105	0.005328 **	0.1151	0.724728
Mc/Ah	−0.24868	0.002098 **	−0.01579	0.968386	−3.8145	0.034771 *
Mc/Ts	−0.26743	0.001260 **	−0.39079	0.337201	−0.4770	0.769002
Ah/Ts	−0.00493	0.938219	−0.54079	0.192420	12.1230	0.000010 ***
Mc/Ah/Ts	−2.04868	0.000243 ***	−3.31579	0.195955	−20.8895	0.056392

Level of statistical significance: * *p* < 0.05, ** *p* < 0.01, *** *p* < 0.001. Mc: *M. communis*, Ah: *A. herba-alba* and Ts: *T. serpyllum*.

## Data Availability

Data are available upon request.

## Supplementary Materials

The following supporting information can be downloaded at: https://www.mdpi.com/article/10.3390/foods11010132/s1, Figure S1: *M. communis* GC analysis chromatogram, Figure S2: *A. herba-alba* GC analysis chromatogram, Figure S3: *T. serpyllum* GC analysis chromatogram, Figure S4: CMI results of the formulation against *B. subtilis*, Figure S5: CMI results of the formulation against *S. aureus*, Figure S6: CMI results of the formulation against *E. coli*.
