# Supplementary material for: Optimized Antibacterial Effects in a Designed Mixture of Essential Oils of Myrtus communis, Artemisia herba-alba and Thymus serpyllum for Wide Range of Applications
Source: Foods. 2022 Jan 5;11(1):132. doi: 10.3390/foods11010132 (PMC8750683; doi:10.3390/foods11010132)
Supplement: Supplementary file 1 [file foods-11-00132-s001.zip › foods-1476238-supplementary.pdf]

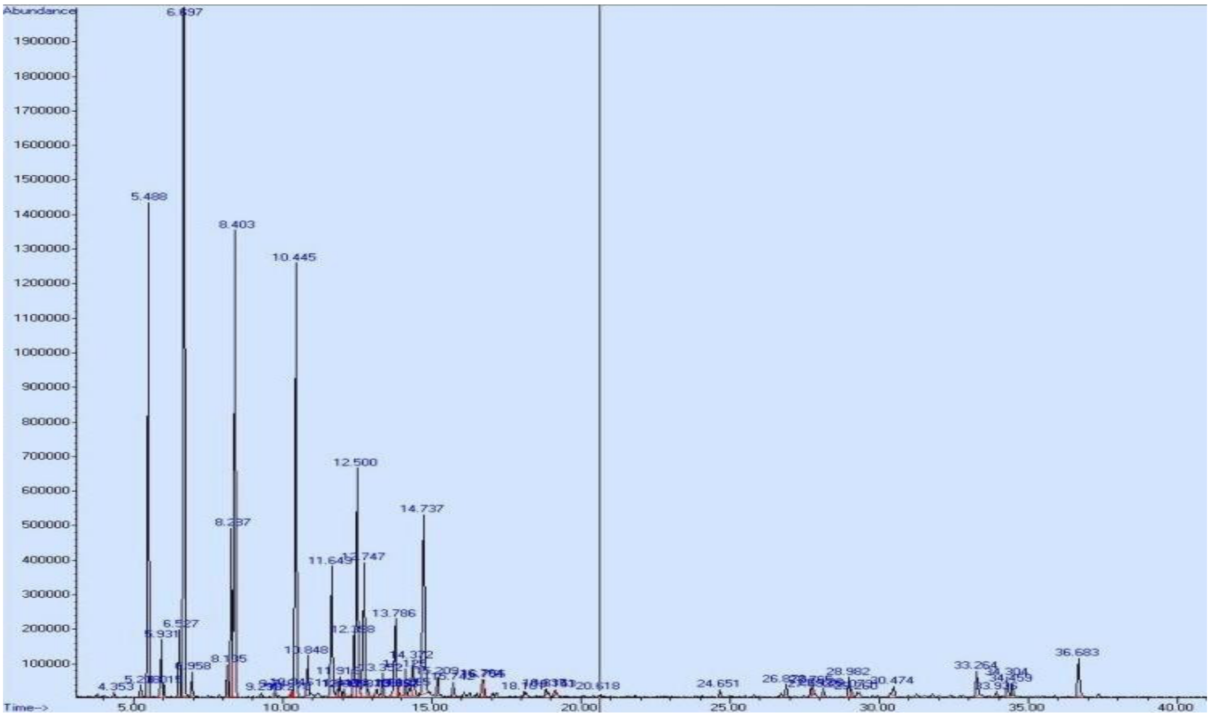

Supplementary Figure S1. *M. communis GC analysis* Chromatogram

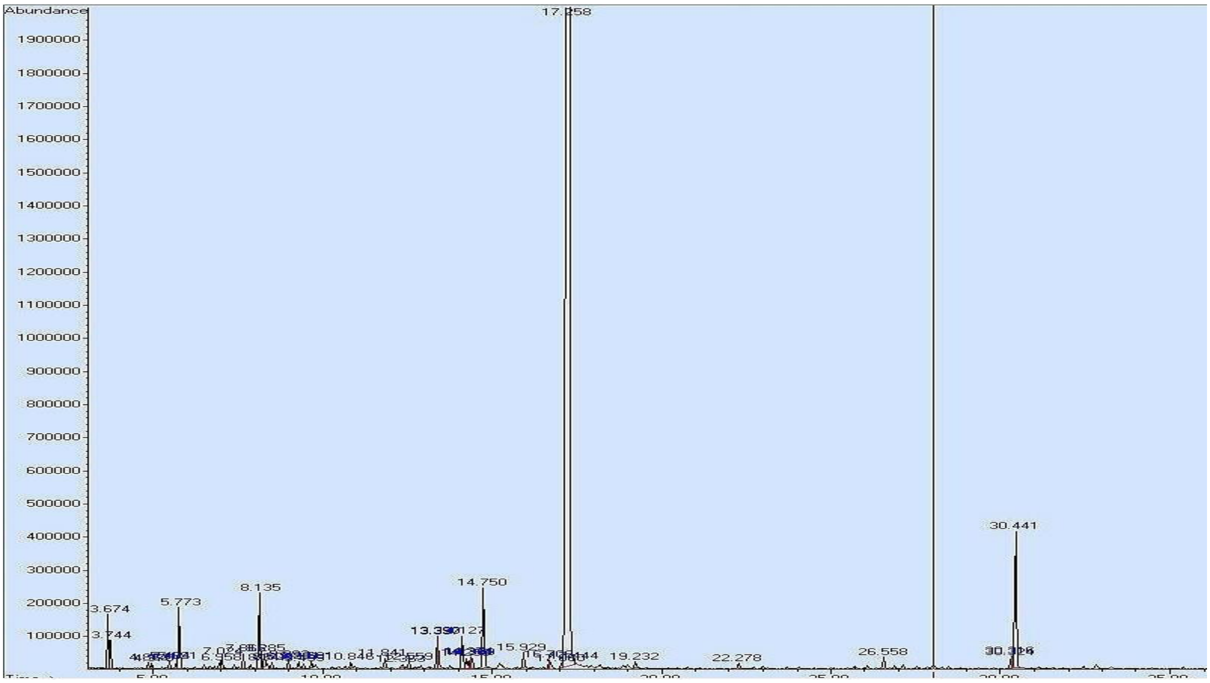

Supplementary Figure S2. *A. herba-alba GC analysis* Chromatogram

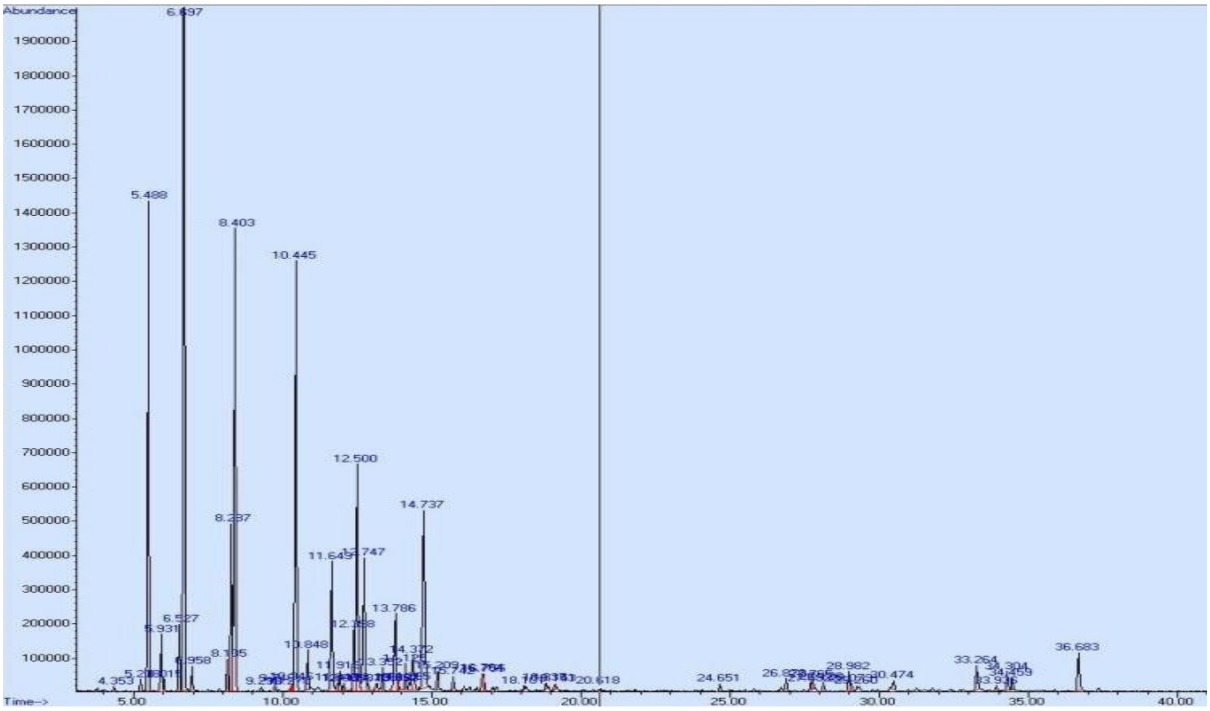

Supplementary Figure S3. *A. herba-alba GC analysis* Chromatogram

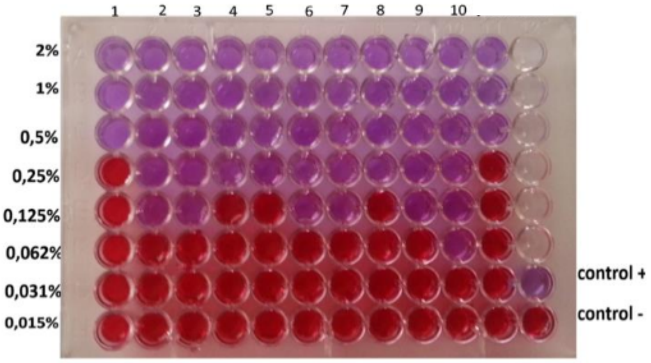

Supplementary Figure S4. CMI results of the formulation against *B. subtilis*

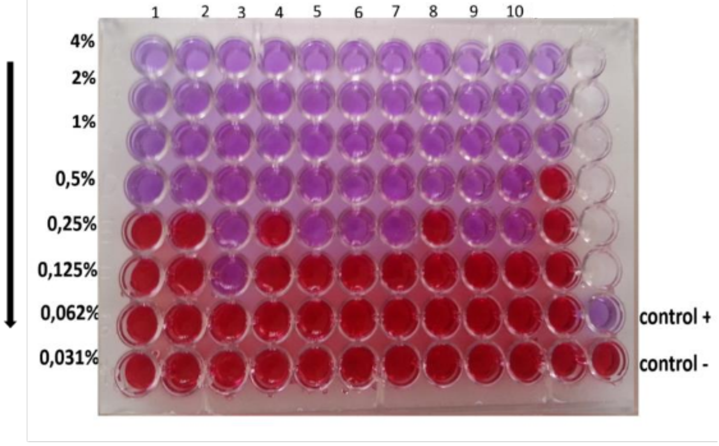

Supplementary Figure S5. CMI results of the formulation against *S. aureus*

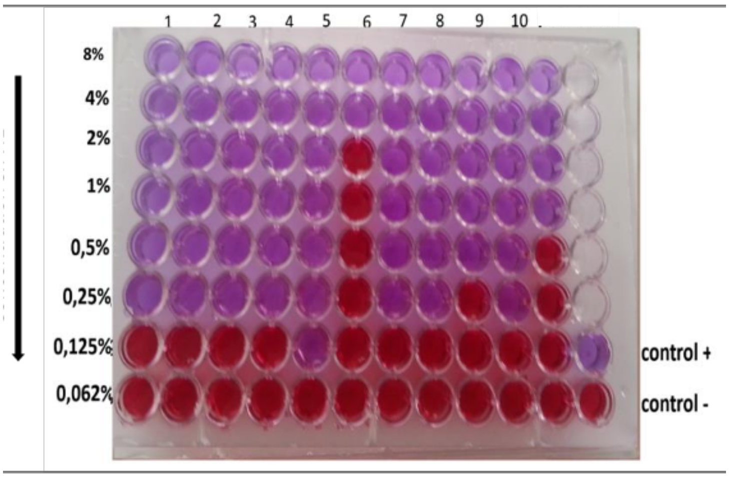

Supplementary Figure S6. CMI results of the formulation against *E. Coli*
